# The Associations of Loneliness and Social Support with Cognitive Impairment among Middle-Aged and Older Individuals with Diabetes

**DOI:** 10.3390/ijerph20031885

**Published:** 2023-01-19

**Authors:** Ella Cohn-Schwartz, Rennie Joshi, Leslie A. McClure

**Affiliations:** 1 Department of Epidemiology, Biostatistics, and Community Health Sciences, Faculty of Health Sciences, Ben-Gurion University, Beer-Sheva 8410501, Israel; 2 Department of Epidemiology and Biostatistics, Drexel University Dornsife School of Public Health, Philadelphia, PA 19104, USA

**Keywords:** loneliness, cognition, aging, living arrangements, support, friends, family, diabetes

## Abstract

Diabetes mellitus is a chronic disease with significant morbidity and mortality and it is associated with poor cognitive performance in later life. This study seeks to determine the relationship between social support and cognitive function among participants with type 2 diabetes mellitus (T2DM). We used data from the Reasons for Geographic and Racial Differences in Stroke (REGARDS) study, including participants with T2DM aged 45 and older (n = 4821). We examined different aspects of perceived social support, measured as structural social support (e.g., marital status), functional social support (having a caregiver in case of sickness or disability), and loneliness. We examined cognitive functioning using a six-item screener. Our results indicate that adults who felt lonely for 5–7 days per week had almost double the odds of cognitive impairment compared to those who didn’t feel lonely. These results suggest that among middle-aged and older individuals with T2DM, interventions targeting lonely adults and which aim to reduce loneliness may combat some of the risks of cognitive decline.

## 1. Introduction

The population is aging; older people comprise a growing segment of the population and their numbers are only expected to increase. Indeed, between 2015 and 2050, the proportion of the world population older than 60 years will nearly double from 12% to 22% [1]. In the United States (U.S.), adults aged 65 or above constitute 16.5% of the general population [2]. Increased longevity brings with it opportunities for successful and healthy aging; however, the degree to which older adults can enjoy these opportunities depends to a large extent on their cognitive health [1]. Cognitive dysfunction is common in older age and constitutes a major public health concern worldwide as a result of global increased life expectancy and population aging [3]. Cognitive function is important for diverse life domains, and poor cognitive function is associated with reduced physical health, loss of independence, depression, and even mortality [4,5,6,7]. In addition, older people with cognitive impairment face an increased risk of developing dementia [8]. 

Research has shown that diabetes constitutes a major risk factor for cognitive dysfunction [9,10,11]. Diabetes mellitus is a chronic disease with significant morbidity and mortality. It can result in long-term damage, dysfunction, and failure of organs, including retinopathy with potential loss of vision, nephropathy leading to renal failure, diabetic gangrene, and cardiovascular and cerebrovascular disease [12]. Diabetes becomes more common with increasing age [13,14]. In 2019, the estimated number of people with diabetes aged 65–99 years was 135.6 million worldwide, constituting 19.3% of older adults, and this number is expected to increase to 276.2 million by 2045 [14]. In the U.S. these numbers are even higher, with diabetes found among 25% of adults aged 65+ in the U.S. and among 16% of those aged 45–64 [15]. Among all individuals with diabetes, about 90–95% have type 2 diabetes mellitus (T2DM) which encompasses individuals who have insulin resistance and usually have relative insulin deficiency [12]. T2DM is a worldwide epidemic, with growing prevalence that is expected to continue to increase over time [14]. These data point not only to the significant increase in diabetes among the aging population, but also to the inevitable public health challenges this increase will bring. Older adults with diabetes are more likely to experience an accelerated rate of cognitive dysfunction and are more likely to progress to dementia [9,10]. Prospective studies indicate that, compared to people without diabetes, individuals with diabetes have a 1.4- to 1.8-fold greater risk of cognitive impairment and are at an increased risk for accelerated cognitive decline [10,16,17], possibly due to the chronic exposure of the brain to high levels of glucose or due to insufficient insulin action in the brain [10].

It is therefore imperative to investigate factors that can attenuate the negative cognitive implications of diabetes. One meaningful, yet under-studied, factor that could help mitigate the impact of diabetes on cognitive function is the social connections of older adults. Low social support and loneliness have long been identified as harmful to cognitive function at older ages [18,19,20,21]. The cognitive enrichment framework explains this by proposing that a socially enriched environment can help preserve and improve cognitive function in older ages [22]. This approach maintains that the brains of older adults can adapt to compensate for neural changes that occur late in life [23] and that this adaptation is influenced by the social context and personal behavior of individuals. More specifically, interactions with other people can act as a form of mental stimulation, as proposed by the “use it or lose it” hypothesis [24]. 

Social support can be divided into functional and structural aspects of support [25]. Structural aspects concern factors such as the number of close relationships one has, living with someone, and being partnered. Functional aspects are related to the quality of ties, such as the availability of support in case of need [26]. Feelings of loneliness can also be conceptualized as a form of functional support, since they concern the perceived quality and adequacy of one’s interpersonal relationships [27]. Structural social support could be more strongly related to cognitive performance since having someone to interact with, regardless of the quality of the relationship, could be cognitively stimulating [28,29]. 

However, existing studies on the associations of social support and loneliness with cognitive function have been carried out among a generally older population. These studies have not focused on individuals with T2DM and did not take the presence or absence of T2DM into account. As such, it is particularly important to understand the association between social support and cognitive function among individuals with T2DM, who face a greater risk of cognitive decline. This need is further emphasized since individuals with T2DM tend to have fewer sources of social support than the general population [30] and to feel greater loneliness [31,32], a difference which can be exacerbated as they grow older and face greater losses of their sources of support [33]. The current study will advance knowledge in this field by focusing specifically on the social support, loneliness experiences, and cognitive function of older individuals with T2DM. 

The current study will fill the aforementioned gaps by examining the associations of social support and loneliness of middle-aged and older people with T2DM with cognitive function. We will examine different aspects of perceived social support, measured as structural social support (e.g., number of close relatives and friends), functional social support (having a caregiver in case of sickness or disability), and loneliness. We hypothesize that: (1) participants with more social support will have lower odds of cognitive impairment than those with less social support; and (2) the association will be stronger for measures of structural support (number of friends, being partnered, living with someone) compared to functional support (availability of a caregiver) and loneliness. 

## 2. Methods

### 2.1. Data and Participants

Data from the Reasons for Geographic and Racial Differences in Stroke (REGARDS) study were used. Details of the REGARDS study have been published elsewhere [34]. In brief, REGARDS is a cohort of English-speaking, community-dwelling, Black and White adults who are aged 45 and older and who lived in the 48 contiguous U.S. at enrollment, between 2003–2007 [34]. The REGARDS study was designed to investigate racial and regional variations in stroke mortality, and oversampled Black individuals and people living in the U.S. stroke buckle (coastal regions of North Carolina, South Carolina, and Georgia) and the rest of the stroke belt (remaining areas of North Carolina, South Carolina, and Georgia; and Alabama, Arkansas, Louisiana, Mississippi, and Tennessee). The institutional review boards at participating institutions approved the study protocol, and all participants provided written informed consent [34]. Information about social support, socio-demographic factors, BMI, and physical exercise was obtained via a computer- assisted telephone interview and an in-person visit. [34]. A brief in-person exam, including blood pressure and physical measurements and blood and urine samples, was conducted approximately 3–4 weeks after the telephone interview. The sample for the current study included all participants with diabetes, measured as self-reported or elevated glucose (fasting glucose ≥126/non-fasting glucose ≥200) at their baseline in-home visit and had full information on the study variables (n = 4812). 

### 2.2. Variables

Cognitive impairment. The Six-Item Screener (SIS) was used for assessment of cognitive impairment. The SIS is a test of global cognitive function that assesses recall and temporal orientation [35,36]. As REGARDS has done in other studies, we dichotomized the score of the SIS into an outcome of cognitively impaired or intact. A score of four or fewer correct answers out of a range of 0–6 indicated cognitive impairment [37,38]. The SIS has been validated against the Mini-Mental State Examination (MMSE), other cognitive measures, and diagnoses of dementia- and nondementia-related cognitive impairment [35]. 

Social support. Functional support was measured with one item of care during illness or disability. Participants were asked, “If you had a serious illness or became disabled, do you have someone who would be able to provide care for you on an ongoing basis?” This item was dichotomized as care during illness or disability vs. no one to care during illness or disability. Loneliness was measured by asking participants how often they felt lonely during a typical week, with response options being: less than a day, 1–2 days, 3–4 days, and 5–7 days. Structural support was measured using three items. Partnered status was a dichotomous variable based on whether participants were married or in a marriage-like relationship vs. divorced, widowed, separated, or never been married. Number of adults in the household was examined by asking participants, “Not counting yourself, how many adults, age 18 or older currently live in the same household with you?” Because of limited variation in this item, we divided it into tertiles: 0 other adults in household, 1 other adult in household, and 2+ other adults in household [26]. Frequency of contacts was assessed by asking participants how many friends or relatives they see at least once a month. 

Covariates. The analyses also took into account potential confounders due to prior evidence regarding their association with cognitive impairment: age (continuous), gender (male or female), education (less than high school, high school, some college, or college diploma), income (less than $20,000/year, $20,000–$35,000/year, $35,000–$75,000/year, or over $75,000/year), race (White or Black), region (non-belt/buckle, stroke belt, or stroke buckle), body mass index (BMI)(underweight, normal, overweight, or obese), and exercise (weekly exercise or no weekly exercise). 

### 2.3. Data Analysis

Descriptive statistics are provided to characterize the sample included in the analysis, overall and by cognitive impairment status. Bivariate analyses examined whether participants who are impaired differ from those without impairment in relation to the study variables; t-tests were used to measure the associations with continuous variables and chi-square tests for categorical or dichotomous variables. We used a hierarchical regression model to examine whether there is an association between the social support items and cognitive impairment among participants with diabetes. All models included the potential confounders described above. We first entered marital status, loneliness, and friends or relatives seen monthly. In model 2, we added the number of household adults, and in model 3, we added having a caregiver in case of sickness or disability. 

## 3. Results

Table 1 presents the baseline characteristics for the entire sample (n = 4812) and by cognitive status. Of the whole sample, 10.7% were identified as cognitively impaired, with a score of 4 or less on the SIS. This percentage is slightly higher compared to the 8% rate of cognitive impairment found in the general population of REGARDS participants [9,37]. Over half of the participants were married, with the remainder predominantly being widowed. Among participants with diabetes who were cognitively impaired, a statistically significantly smaller proportion were married adults. Participants with diabetes who were not impaired reported having 7.6 friends or relatives seen at least monthly, while those who were impaired reported 6.8 (*p* = 0.026). Less than a third of the participants who were not impaired reported living alone, while among those who were cognitively impaired, a higher proportion lived alone (27% versus 33%, respectively). Impaired participants also reported feeling lonely nearly twice as frequently as non-impaired participant (7% vs. 4% for 5–7 days weekly). The two groups did not significantly differ in their frequency of having a caregiver in case of sickness or disability. 

Table 1 also shows that the sample was comprised of more women than men; this did not differ by cognitive status. Being cognitively impaired was associated with having significantly lower educational attainment. The average age of the sample was 65.6 (SD = 8.6) and participants who were cognitively impaired were older compared to adults who were not impaired (average age 69 (SD = 9.0) vs. 65 (SD = 8.5). Participants with T2DM and cognitive impairment had a lower income and were more frequently Black compared to non-impaired participants, but the two groups did not differ in relation to their region of residence. The two groups also differed in regards to medical and lifestyle factors, with the participants with T2DM and cognitive impairment being less frequently obese and reporting less frequent weekly exercise. 

Table 2 presents the hierarchical multivariable regression assessing the association between cognitive impairment and the social support variables. All models included potential confounders: gender, age, education, income, race, region, BMI, and exercise. The first model included the structural variables of marital status and number of friends and relatives seen weekly, and the functional aspect of loneliness. The results indicated that being married or having more close friends and relatives were not associated with cognitive impairment. Loneliness was associated with higher odds of cognitive impairment, such that participants with T2DM who reported feeling lonely for 5–7 days in a week had almost twice the odds of cognitive impairment compared to those who reported feeling lonely less than one day per week (OR = 1.91, *p* = 0.002, CI: 1.28–2.86). The structural support indicator number of adults living in the household, added in model 2, was not statistically significantly associated with cognitive impairment. The third model, which included the functional support indicator of having a caregiver in case of sickness or disability, was also not associated with cognitive impairment. In the full model, being lonely for 5–7 days per week remained significantly associated with cognitive impairment, and the magnitude of the association was unchanged (OR = 1.92, *p* = 0.002, CI: 1.28–2.87).

## 4. Discussion

Given the increased risks of cognitive dysfunction faced by individuals with T2DM, and the increased prevalence of T2DM among an aging population, it is crucial to find ways to help this population in the face of their greater vulnerability. An investigation of middle-aged and older adults is particularly warranted as the number of older adults living with diabetes is rapidly increasing, as are the risks of cognitive dysfunction when adults reach later life. The current study examined both structural support, such as the number of close friends and relatives and marital status, and functional support, namely the availability of a caregiver and loneliness experiences. Our results identified loneliness as associated with cognitive impairment among participants with T2DM. 

As we hypothesized, cognitive impairment among participants with T2DM was associated with social support. However, unlike our second supposition, cognitive impairment was not associated with the structural aspects of support but rather with loneliness, which describes a more subjective perception of the quality of one’s social environment. In particular, adults who reported feeling lonely at the highest frequency, 5–7 days per week, had almost twice the odds of cognitive impairment compared to those who reported feeling lonely less than one day per week. These findings suggest that it isn’t necessarily the presence or absence of others that influences impairment, but rather that the perception of loneliness is more relevant. Although previous investigations pointed to the importance of having someone to interact with, our findings emphasize that having a support network that matches one’s expectation should also be measured and focused upon in future studies and interventions. Various mechanisms may underlie this association. For example, people with T2DM who are very lonely may have poorer diabetes self-management, less glycemic control, and reduced treatment adherence, and they may adopt a less active lifestyle, which are crucial aspects of diabetes management [39,40]. They could have a less available social support network, which would impede their ability to implement and sustain key behaviors in order to control their blood glucose, leading to worse cognitive health outcomes [39,40,41,42,43,44]. Accordingly, frequent loneliness among individuals with diabetes has been associated with low medication adherence and not exercising regularly [31]. Loneliness may also affect cognition through depression. People with T2DM experiencing loneliness may also be depressed, as loneliness is a major risk factor for depression and has been shown to be predictive of the development of depressive symptoms and depression [45]. For example, a longitudinal study among 285 adults aged 60–90 found that loneliness affected the prognosis of depression and the severity of its symptoms and was associated with poorer outcomes after two years [46]. In turn, being depressed is an emotional state which can harm cognitive function and lead to cognitive impairment [22,47]. Future research could examine depression as a mediator in the association of social support and loneliness among people with T2DM. 

These results can provide a foundation on which to build better-targeted and more effective interventions aimed at improving the cognitive function of older adults with T2DM. These insights can inform practitioners to especially focus on perceptions of loneliness among individuals with T2DM. Interventions for middle-aged and older individuals with T2DM should target those who suffer from frequent loneliness, which may possibly mitigate some of the risk of cognitive decline. It is important to identify lonely adults by asking them whether they feel lonely, isolated, or left out and how often they experience those feelings. Various interventions, such as group therapy and internet or telephone-based peer support, may be useful in alleviating some of the loneliness that may plague individuals with T2DM as they age [48,49].

The current findings should be interpreted in light of the study’s limitations. Cognitive function was measured using a single indicator that assesses global cognitive performance. Although the Six-Item Screener has been shown to be effective in capturing cognitive impairment [35], it is a single score and therefore cannot differentiate between various aspects of cognitive function [18]. Thus, future research should examine the associations of social support and different aspects of cognition. Additionally, this was a cross-sectional study, and therefore, it was not possible to determine the temporality between social support and cognitive impairment, as it is possible that, in addition to the effect of loneliness on cognitive impairment, individuals with T2DM who are cognitively impaired may experience deteriorations in their feelings of loneliness. Therefore, future research should take the issue of reverse causality into account, as well as consider examining cognitive functioning over time. An additional issue is that the data was collected between 2003 and 2007, while the COVID-19 pandemic changed many facets of society, relations, and well-being. Thus, future studies should examine the current research questions both during and after the pandemic.

The research described herein also has strengths. The cohort is well-described and includes a large sample of Black and White adults aged 45 and older. The Six-Item Screener has been shown to be a good marker of cognitive functioning. Further, a number of potential confounders were measured and included in the analyses.

To conclude, diabetes mellitus is a chronic disease with significant morbidity and mortality and is associated with poor cognitive performance and cognitive decline [9,16]. It is thus imperative to find factors that can attenuate the negative cognitive implications of diabetes. The results of the current study indicate that among people with T2DM, frequent loneliness was associated with nearly double the odds of having cognitive impairment. These findings indicate that combatting loneliness among middle-aged and older individuals with diabetes may help improve their cognitive function. Because T2DM is related to an increased risk of cognitive impairment, discovering the association of social factors with cognitive functioning within the context of this disease may lead not only to better understanding of individuals with diabetes but also to the development of targeted interventions to enhance their cognitive functioning and possibly their everyday functioning and quality of life as a consequence.

## Figures and Tables

**Table 1:** Sample characteristics overall and by cognitive status among REGARDS participants.

Characteristics	All Subjects (N = 4812)	Intact Cognitive Status (N = 4297, 89.3%)	Impaired Cognitive Status (N = 515; 10.7%)	*p*-Value
	N (%)\M (SD)	N (%)\M (SD)	N (%)\M (SD)	
**Social support**				
Marital Status:				**<0.001**
Married	2643 (54.9)	2400 (55.9)	243 (47.2)	
Single	275 (5.7)	242 (5.6)	33 (6.4)	
Other	1894 (39.4)	1655 (38.5)	239 (46.4)	
No. of close friends or relatives seen at least monthly	7.5 (7.7)	7.6 (7.7)	6.8 (7.5)	**0.026**
No. of other adults in household:				**0.024**
0	1345 (28.0)	1175 (27.3)	170 (33.0)	
1	2575 (53.5)	2316 (53.9)	259 (50.3)	
2+	892 (18.5)	806 (18.8)	86 (16.7)	
Loneliness:				**<0.001**
<1 day	3549 (73.8)	3210 (74.7)	339 (65.8)	
1–2 days	809 (16.8)	704 (16.4)	105 (20.4)	
3–4 days	246 (5.1)	211 (4.9)	35 (6.8)	
5–7 days	208 (4.3)	172 (4.0)	36 (6.9)	
Having a caregiver in case of sickness or disability:				0.457
Yes	4144 (86.1)	3706 (86.3)	438 (85.1)	
No	668 (13.9)	591 (13.8)	77 (15.0)	
**Demographics**				
Gender:				
Female	2543 (52.9)	2260 (52.6)	283 (55.0)	0.311
Male	2269 (47.1)	2037 (47.4)	232 (45.0)	
Education:				**<0.001**
Less than High School	901 (18.7)	718 (16.7)	183 (35.5)	
High School	1361 (28.3)	1216 (28.3)	145 (28.2)	
Some College	1270 (26.4)	1174 (27.3)	96 (18.6)	
College Diploma	1280 (26.6)	1189 (27.7)	91 (17.7)	
Age	65.6 (8.6)	65.2 (8.5)	68.6 (9.0)	**<0.001**
Income:				**<0.001**
Less than $20,000/year	1186 (24.7)	1013 (23.6)	173 (33.6)	
$20,000–$35,000/year	1316 (27.4)	1160 (27.0)	156 (30.3)	
$35,000–$75,000/year	1282 (26.6)	1203 (28.0)	79 (15.3)	
Over $75,000/year	459 (9.5)	439 (10.2)	20 (3.9)	
Refused	569 (11.8)	482 (11.2)	87 (16.9)	
Race:				**<0.001**
Black	2792 (58.0)	2401 (55.9)	391 (75.9)	
White	2020 (42.0)	1896 (44.1)	124 (24.1)	
Region:				0.289
Non-Belt/Buckle	2043 (42.5)	1833 (42.7)	210 (40.8)	
Stroke Belt	1710 (35.5)	1511 (35.2)	199 (38.6)	
Stroke Buckle	1059 (22.0)	953 (22.2)	106 (20.6)	
BMI:				**<0.001**
Normal	518 (10.8)	432 (10.1)	86 (16.7)	
Overweight	1447 (30.1)	1277 (29.7)	170 (33.0)	
Obese	2847 (59.2)	2588 (60.2)	259 (50.3)	
Weekly exercise:				**0.041**
≥4 times	1149 (23.9)	1020 (23.7)	129 (25.1)	
1–3 times	1678 (34.9)	1524 (35.5)	154 (29.9)	
None	1985 (41.3)	1753 (40.8)	232 (45.1)	

BMI = body mass index. *p*-values for categorical variables provided from a chi-squared test statistic and for continuous variables provided from a t-test statistic calculated for each variable by cognitive status. *p*-values in bold indicate values that are significant at α = 0.05.

**Table 2:** Multivariable models of association between cognitive impairment and social variables among REGARDS participants (n = 4812).

	Model 1: Marital Status, Lonely Days, Friends or Relatives Seen Monthly				Model 2: Model 1 + No. of Household Adults				Model 3: Model 2 + Having a Caregiver in Case of Sickness or Disability			
Parameter	OR	LCI	UCI	*p*	OR	LCI	UCI	*p*	OR	LCI	UCI	*p*
Marital Status												
Unmarried vs. Married	0.99	0.79	1.23	0.9207	0.94	0.72	1.232	0.6605	0.94	0.72	1.24	0.6698
Lonely days per week												
1–2 days vs. less than 1 day	1.25	0.98	1.60	0.0720	1.25	0.98	1.603	0.0711	1.26	0.98	1.61	0.0700
3–4 days vs. less than 1 day	1.29	0.87	1.92	0.2030	1.30	0.87	1.927	0.2003	1.30	0.87	1.93	0.1974
5–7 days vs. less than 1 day	**1.91**	1.28	2.86	0.0015	**1.91**	1.28	2.849	0.0016	**1.92**	1.28	2.87	0.0016
Friends or relatives seen monthly	0.99	0.98	1.00	0.1101	0.99	0.98	1.003	0.1155	0.99	0.98	1.00	0.1140
No. of household adults												
0 vs. 2+					1.21	0.88	1.674	0.2482	1.21	0.88	1.68	0.2458
1 vs. 2+					1.14	0.87	1.490	0.3338	1.14	0.87	1.49	0.3320
Having a caregiver in case of sickness or disability									0.98	0.74	1.29	0.8725
Nagelkerke R^2^		0.0080					0.014				0.115	

Notes. OR = odds ratio. *p* = *p*-value. Adjusted for gender, age, education, income, race, region, BMI, and exercise. ORs in bold have CI’s which do not overlap a null value.

## Data Availability

Data is available by collaborating with REGARDS investigators, through contact with: REGARDSAdmin@uab.edu. For additional information see: https://www.uab.edu/soph/regardsstudy/researchers (accessed on 1 January 2023).
